# Effects of Pranoprofen on Aqueous Humor Monocyte Chemoattractant Protein-1 Level and Pain Relief During Second-Eye Cataract Surgery

**DOI:** 10.3389/fphar.2018.00783

**Published:** 2018-07-17

**Authors:** Yinglei Zhang, Yu Du, Yongxiang Jiang, Xiangjia Zhu, Yi Lu

**Affiliations:** ^1^Department of Ophthalmology, Eye and Ear, Nose, and Throat Hospital, Fudan University, Shanghai, China; ^2^Eye Institute, Eye and Ear, Nose, and Throat Hospital, Fudan University, Shanghai, China; ^3^Key Laboratory of Myopia, Ministry of Health, Shanghai, China; ^4^Shanghai Key Laboratory of Visual Impairment and Restoration, Shanghai, China

**Keywords:** cataract surgery, pain, pranoprofen, MCP-1, ECLE rat model

## Abstract

The aim of our present study is to evaluate the efficacy of pranoprofen eye drops as pain relief during sequential second-eye cataract surgery and to investigate the possible mechanism. Seventy-six patients scheduled for bilateral sequential cataract surgery were randomly assigned to two groups: (1) treatment group (administered pranoprofen eye drops), or (2) control group (administered artificial tears). Preoperative anxiety and intraoperative pain were assessed. Monocyte chemoattractant protein 1 (MCP-1) in the aqueous humor was measured with a suspension cytokine array. An extracapsular lens extraction model was established in the Wistar rat and the MCP-1 concentrations were measured with an enzyme-linked immunosorbent assay. We found that in the control group, the pain scores were significantly higher during second-eye surgery than during first-eye surgery (both scores *P* < 0.001). In the treatment group, there was no significant difference in the pain scores during first-eye and second-eye surgery (both scores *P* > 0.1). The pain during second-eye surgery was significantly lower in the treatment group than in the control group (both scores *P* < 0.01). And in the 1-week and 6-week interval subgroups, the pain scores during second-eye surgery were significantly lower in the treatment group than the control group (*P* = 0.047 and *P* = 0.035, respectively). While the second-eye MCP-1 level was significantly lower after a 1-week interval in the treatment group than in the control group (*P* = 0.012), but did not differ significantly after a 6-week interval (*P* > 0.1). A parallel trend in the MCP-1 concentration was detected in the rat model. In conclusion, the preoperative administration of pranoprofen eye drops reduced the perceived pain during second-eye cataract surgery, especially when performed after 1-week and 6-week intervals between the first-eye and second-eye surgery. MCP-1, a pain-related cytokine, was associated with the pain-relief mechanism of pranoprofen when second-eye surgery was performed 1 week after second-eye surgery.

## Introduction

Phacoemulsification with intraocular lens (IOL) implantation under topical anesthesia is widely performed to treat cataract (Fichman, 1996). Cataract surgery is an invasive procedure, breaking the blood–aqueous humor barrier, and increases prostaglandin production. Consequently, surgery is associated with complications such as pain, hyperemia, and cystoid macular edema (Nardi et al., 2007; Bucci and Waterbury, 2011). Notably, a subtle increase in pain during second-eye surgery relative to that during first-eye surgery has been reported (Cheng et al., 2006; Tan et al., 2011; Ursea et al., 2011). Intraoperative pain might reduce the patient’s willingness to cooperate during surgery and their satisfaction with surgery, and this increases the difficulty of surgery. Therefore, the requirement for pain relief increases during second-eye surgery. Nonsteroidal anti-inflammatory drugs (NSAIDs) exert potent analgesic effects by inhibiting the biosynthesis of prostaglandins, which normally sensitize the pain nerve endings (McCormack and Brune, 1991; Sher et al., 1993). Two clinical trials have demonstrated that bromfenac eye drops reduce ocular inflammation and pain after cataract surgery (Donnenfeld et al., 2007). In other ophthalmic settings, NSAIDs have also been used to reduce pain after photorefractive keratectomy (PRK) (Faktorovich and Melwani, 2014). The preoperative administration of NSAIDs has also been shown to disrupt the inflammatory cascade and prevent the synthesis and release of prostaglandins (Rowen, 1999). However, the effects of preoperative NSAIDs on pain relief during sequential second-eye cataract surgery are unclear.

In our previous study, we reported for the first time that the concentration of monocyte chemoattractant protein 1 (MCP-1), a pain-related inflammatory cytokine, increases in the aqueous humor during sequential second-eye surgery relative to that during first-eye surgery (Zhu et al., 2015). We determined the inflammatory status in the second eye, which might be induced by the first-eye surgery, and may increase pain perception. Does the preoperative use of NSAIDs affect the level of MCP-1 in the second eye?

In this study, we tested the hypothesis that the preoperative administration of pranoprofen ophthalmic solution reduces ocular pain during sequential second-eye surgery. The role of MCP-1 in the pain relief afforded by pranoprofen was also investigated and then verified in the extracapsular lens extraction (ECLE) rat model.

## Materials and Methods

This prospective, single-blind, randomized controlled study was approved by the Ethics Committee of the Eye and ENT Hospital, Fudan University, Shanghai, China. The study was registered at www.clinicaltrials.gov (registration number: NCT02182921). Written consent was obtained from all the patients after they were informed of the nature and possible consequences of the study. All procedures adhered to the tenets of the Declaration of Helsinki.

### Patients

From October 2016, we screened all the consecutive patients scheduled for bilateral sequential phacoemulsification with IOL implantation under topical anesthesia at the Eye and ENT Hospital of Fudan University. All eligible patients underwent bilateral cataract surgery, and sequential second-eye surgery was performed within 8 weeks of first-eye surgery. Patients with any of the following were excluded: any nerve blockage or general anesthesia; any pathological features other than cataract; a history of ocular trauma or surgery; a history of allergic reaction to NSAIDs; or a history of diabetes.

The enrolled patients were randomly assigned to the control group (*N* = 38) or the treatment group (*N* = 38). Patients in the treatment group were administered pranoprofen eye drops three times in 1 h before second-eye surgery. Patients in the control group received artificial tears three times in 1 h before second-eye surgery, as a placebo. Clinical data, including age, sex, interval between first-eye and second-eye surgery, preoperative visual acuity, axial length, the Lens Opacities Classification System version III grade, ultrasound power, and ultrasound time, were recorded. In each group, the patients were divided into eight subgroups according to the interval between the first-eye and second-eye surgery.

All participants were blinded to the function of the preoperative eye drops they received during the whole study. The researchers and statisticians had full access to the data, but none of them had any financial interest in this study.

### Surgical Procedures and Aqueous Humor Acquisition

In both groups, aqueous humor (100 μl) samples were obtained during first-eye and second-eye surgery through the paracentesis site, followed by an injection of viscoelastic material (DisCoVisc; Alcon Laboratories, Inc., Fort Worth, TX, United States). A 2.6 mm temporal clear corneal incision was then made. Hydrodissection, chopping, nucleus rotation, and phacoemulsification were performed after a 5.5 mm continuous curvilinear capsulorhexis was performed. A foldable IOL was implanted with a dedicated injector. After the aspiration of any residual viscoelastic material, the incision was hydrated with balanced salt solution and checked for water tightness. During surgery, the pupil was sufficiently dilated without any intraoperative iris prolapse or bite. All cataract surgery was uneventful and performed by the same surgeon (YL). The aqueous humor samples were immediately stored at −80°C until cytokine analysis.

### Anxiety and Pain Evaluation

Preoperative anxiety was evaluated 10 min before the first-eye and second-eye surgery using the validated simplified State–Trait Anxiety Inventory (STAI, six questions) and a visual analog scale (VAS) for anxiety, which was presented as a numbered line ranging from 0 (no anxiety) to 10 (unbearable anxiety). Postoperative pain was evaluated immediately after surgery using a VAS for pain, which was presented as a numbered line ranging from 0 (no pain) to 10 (unbearable pain), and the Wong–Baker FACES Pain Rating Scale (WBS), which comprises six faces ranging from a happy face for no pain (score = 0) to a crying face for worst pain (score = 10). After second-eye surgery, the patients were also asked to compare the severity of pain experienced in the two procedures.

### Animal Experiments

The animal experiments were approved by the Ethics Committee for Animal Studies at the Eye and ENT Hospital of Fudan University, and the experimental procedures conformed to the ARVO Statement for the use of animals in research.

The experiments were performed in male Wistar rats (160 ± 20 g, 2 months old; SLAC Laboratory Animal Co., Ltd., Shanghai, China). All animals were maintained in cages under a 12 h light–dark cycle at 21 ± 2°C, with a regular diet, for 3 weeks. An intraperitoneal injection of 10% chloral hydrate was given to the rats to induce general anesthesia. Their pupils were then dilated with tropicamide phenylephrine eye drops (Santen Pharmaceutical, Japan). In the ECLE model, a corneal incision was made with a 15° stab knife, and the aqueous humor was collected with a 20 μl capillary tube. A viscoelastic material (hyaluronic acid; Qisheng Biologic Preparation, China) was then injected into the anterior chamber. The anterior capsule was punctured with a 1 ml needle. The corneal incision was then extended to approximately 90° with Vannas scissors, followed by hydrodissection and lens removal. Finally, the incision was closed with 10–0 sutures, and topical Tobradex ointment (0.3% tobramycin and 0.1% dexamethasone; Alcon Laboratories) was administered. After 1 day, 3 days, 1 week, 2 weeks, and 3 weeks, the aqueous humor of the second eye in the control group was collected with the same method and stored at −80°C. In the treatment group, pranoprofen eye drops were administered to the second eye three times in the 1 h before the acquisition of the aqueous humor.

### Measurement of MCP-1 Concentrations

As in our previous study (Zhu et al., 2015), we measured the concentrations of MCP-1 in the human aqueous humor samples with a suspension cytokine array (RayBio; RayBiotech Inc., Norcross, GA, United States), according to the manufacturer’s instructions. After the samples were incubated for 30 min with capture-antibody-coupled magnetic beads, they were washed three times in a Bio-Plex Pro wash station. A biotinylated detection antibody was then added to each well and incubated for 1 h in the dark. The captured analyte was detected by the addition of streptavidin–phycoerythrin and quantified with a Bio-Plex array reader. The fluorescence intensity of the array dots corresponded to the concentration of MCP-1.

The concentration of MCP-1 in the rat aqueous humor was then measured with an enzyme-linked immunosorbent assay (RayBiotech, Inc.). At each time interval, five model rats were included and only 10 μl of aqueous humor was extracted from each, which were then pooled into one sample. Therefore, the measured concentration represented the average MCP-1 levels in five rats.

### Statistical Analysis

SPSS software version 19.0 (IBM-SPSS, Armonk, NY, United States) was used for all statistical analyses. Categorical values are presented as percentages of patients and continuous variables are presented as means and standard deviations (SD). A paired *t*-test or Student’s *t*-test was used to determine differences in continuous variables. The correlation between pain scores and MCP-1 was determined with Pearson’s bivariate correlation test. All *P-* values were two-sided, and values of *P* < 0.05 were considered statistically significant. Comparisons of the intraoperative pain scores were controlled with Bonferroni’s correction (corrected *P*-values of 0.05/2 = 0.025).

## Results

As indicated in **Table 1**, there were no significant differences in the demographic characteristics of the treatment and control groups (all *P* > 0.05).

**Table 1:** Characteristics of the treatment and control groups.

Parameter	Treatment group	Control group	*P*-value
**Sex (M/F)**	14/24	14/24	–
**Age (years)**	67.6 ± 9.5	66.6 ± 8.2	0.618
**Interval time (days)**	32.3 ± 16.9	31.0 ± 22.2	0.885
**Preoperative BCVA (logMAR)**	0.91 ± 0.61	1.00 ± 0.94	0.635
**AL (mm)**	27.26 ± 4.25	26.95 ± 3.74	0.747
**Nuclear grading**	3.0 ± 0.5	3.0 ± 0.5	0.899
**Ultrasound time(s)**	47.3 ± 25.3	43.6 ± 22.9	0.555
**Ultrasound power (%)**	32.1 ± 12.2	33.0 ± 13.7	0.795

*BCVA, best corrected visual acuity; AL, axial length; logMAR, logarithm of the minimum angle of resolution. Lens Opacities Classification System, version III (LOCSIII) grading.*

### Intraoperative Pain Perception in Patients

**FIGURE 1:**
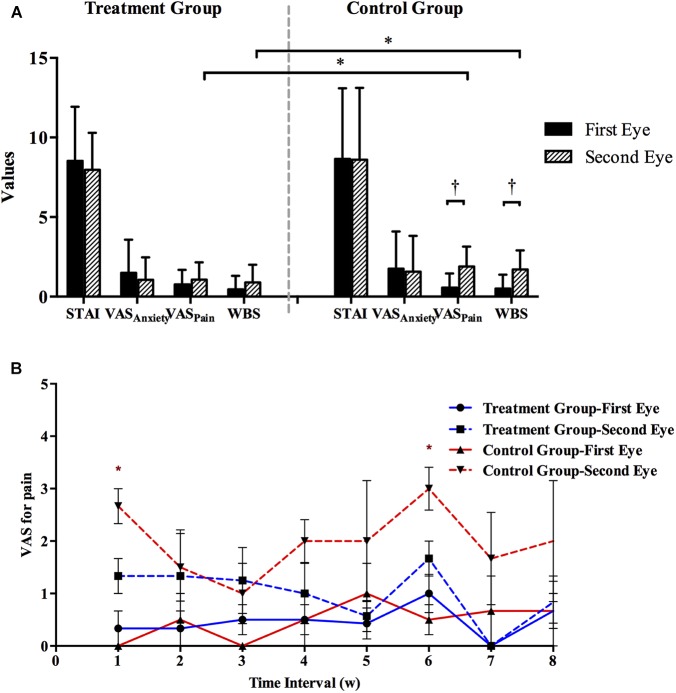
**(A)** Comparison of subjective measures during first-eye and second-eye surgery in the treatment (*N* = 38) and control groups (*N* = 38). The mean interval time between first-eye and second-eye surgeries of patients in treatment and control group was 32.3 ± 16.9 and 31.0 ± 22.2, respectively. ^∗^Bonferroni corrected, *P* < 0.025; ^†^Bonferroni corrected, *P* < 0.025. **(B)** Comparison of VASpain scores (mean with SE) in eight subgroups established based on the interval (weeks) between first-eye and second-eye cataract surgery in the control and treatment groups. ^∗^*P* < 0.05. STAI, State–Trait Anxiety Inventory; VASanxiety, visual analog scale for anxiety; VASpain, visual analog scale for pain; WBS, Wong–Baker FACES Pain Rating Scale; SE, standard error.

In terms of the discomfort perception in the control group, both pain scores (VAS and WBS) were significantly greater during second-eye surgery (1.90 ± 1.24 and 1.70 ± 1.21, respectively) than during first-eye surgery (0.57 ± 0.88 and 0.50 ± 0.88, respectively; both *P* < 0.001; **Figure 1A**). However, in the treatment group, there was no significant difference in the VAS_pain_ or WBS scores between the first-eye (0.77 ± 0.91 and 0.46 ± 0.85, respectively) and second-eye surgery (1.07 ± 1.08 and 0.89 ± 1.11, respectively; both *P* > 0.1, **Figure 1**). Although there was no significant difference in the pain scores during first-eye surgery between the treatment and control groups, the scores for second-eye surgery were significantly lower in the treatment group than in the control group (*P*_VAS_ = 0.004 and *P*_WBS_ = 0.006; **Figure 1A**). Moreover, there were no significant differences between the two groups in either of the anxiety scores before first-eye surgery (*P*_STAI_ = 0.910 and *P*_VAS_ = 0.628) or those before second-eye surgery (*P*_STAI_ = 0.602 and *P*_VAS_ = 0.411). The anxiety scores before the second-eye surgery did not differ from those before first-eye surgery in either group (all *P* > 0.2).

The recruited patients were also divided into eight subgroups based on the interval (weeks) between the first-eye and second-eye cataract surgery, and at least three patients were enrolled in each subgroup. In treatment group, the exact number of patients in each subgroup was 4, 4, 4, 4, 5, 6, 5, and 6, respectively. In control group, the number of patients in each subgroup was 4, 5, 5, 6, 4, 5, 4, and 5, respectively. The pain scores during first-eye surgery in all the subgroups did not differ statistically between the control and treatment groups (all *P* > 0.5; **Figure 1B**). While in the control group, the VAS pain scores during second-eye surgery were greater in 1-week interval (2.67 ± 0.58) and 6-week interval (3.00 ± 0.82) subgroups, and were significantly lower in the treatment group in these two subgroups (1.33 ± 0.58 [*P* = 0.047] and 1.67 ± 0.82 [*P* = 0.035], respectively).

### MCP-1 Concentrations in Patients

**FIGURE 2:**
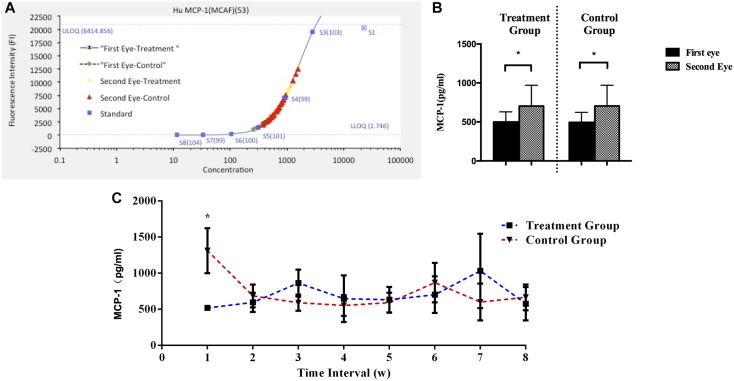
**(A)** Standard curve for MCP-1. **(B)** Aqueous humor MCP-1 concentrations during first- and second-eye surgery in treatment (*N* = 38) and control groups (*N* = 38). The interval time between first-eye and second-eye surgeries in treatment and control group was 32.3 ± 16.9 and 31.0 ± 22.2, respectively. **(C)** Comparison of MCP-1 levels in the second eye in eight subgroups based on the interval (weeks) between first-eye and second-eye cataract surgery in the control and treatment groups. MCP-1, monocyte chemoattractant protein 1. ^∗^*P* < 0.05

In both groups, the aqueous humor MCP-1 levels were significantly greater in second-eye than in first-eye (*P*_Control_ = 0.041 and *P*_Treatment_ = 0.039, paired *t*-test; **Figures 2A,B**). In the second eye, the mean concentration of MCP-1 did not differ significantly between the treatment and control groups (702.70 ± 268.39 and 704.10 ± 265.98 pg/ml, respectively, *P* = 0.983). Then the second-eye MCP-1 levels were significantly lower in the treatment group than in the control group only after a 1-week interval (517.23 ± 29.27 pg/ml vs. control 1310.39 ± 311.46 pg/ml, *P* = 0.012), and no significant differences were detected in the MCP-1 concentrations in other interval subgroups (all *P* > 0.1; **Figure 2C**).

**FIGURE 3:**
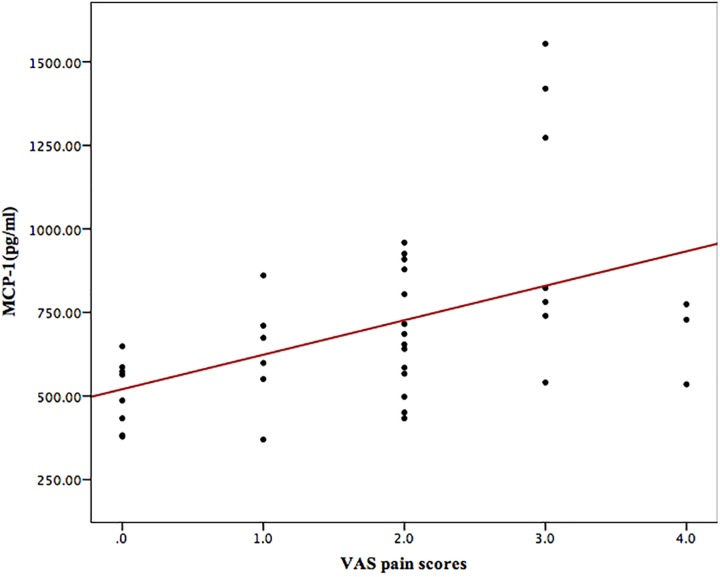
Scatterplot of VASpain scores and aqueous humor MCP-1 concentrations in control subjects (*N* = 38) during the second-eye surgery (correlation: *r* = 0.414, *P* = 0.023, *r*^2^ = 0.171). Pearson’s bivariate correlation analysis was used to determine the relationships between pain perception and aqueous humor cytokines. MCP-1, monocyte chemoattractant protein 1; VASpain, visual analog scale for pain.

Furthermore, the MCP-1 levels across all the subjects in the control group correlated significantly with the VAS_pain_ scores during the second-eye cataract surgery, with a correlation coefficient of *r* = 0.414, *P* = 0.023, *r*^2^ = 0.17 (**Figure 3**).

### MCP-1 Concentrations in Wistar Rats

**FIGURE 4:**
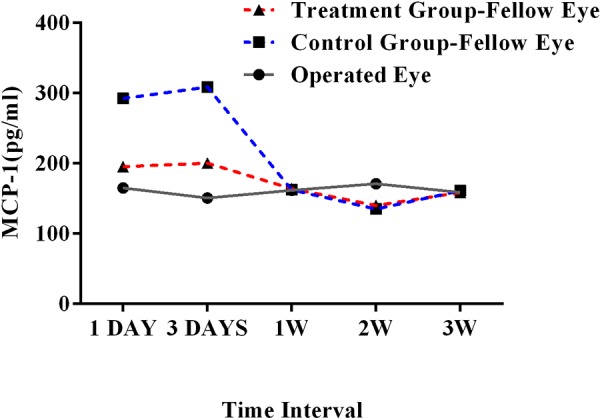
MCP-1 concentration in operated and fellow eyes in treatment and control groups at 1-day, 3-day, 1-week, 2-week, and 3-week intervals in the ECLE rat model. MCP-1, monocyte chemoattractant protein 1; ECLE, extracapsular lens extraction.

In the ECLE rat model, the average MCP-1 levels in the aqueous humor after 1-day and 3-day intervals were 195.36 pg/ml and 200.40 pg/ml, respectively, in treatment group, which were clearly lower than those in the control group (292.40 pg/ml and 308.95 pg/ml, respectively). The MCP-1 concentrations did not differ markedly between the treatment and control groups after a 1-week interval (162.50 pg/ml and 164.25 pg/ml, respectively), 2-week interval (135.09 pg/ml and 140.05 pg/ml, respectively), or 3-week interval (161.25 pg/ml and 158.77 pg/ml, respectively, **Figure 4**).

## Discussion

Topical anesthesia is widely used in cataract surgery, and patient cooperation is crucial during the procedure. A peculiar aspect of phacoemulsification is its symmetry. Previous studies have reported a subtle increase in pain during second-eye cataract surgery relative to that during first-eye surgery. Intraoperative pain might reduce the patient’s willingness to cooperate, increasing surgical difficulty and reducing patient satisfaction (Ursea et al., 2011; Jiang et al., 2015). The perioperative administration of NSAIDs is reported to improve postoperative comfort. However, to the best of our knowledge, no study has examined the pain relief provided by NSAIDs during second-eye cataract surgery. Therefore, in this study, we evaluated the effects of administering pranoprofen eye drops for pain relief during sequential second-eye cataract surgery, and investigated the associated molecular mechanism, which was verified in the ECLE rat model.

This study demonstrates for the first time that the preoperative administration of pranoprofen eye drops significantly reduces the intraoperative pain in sequential second-eye surgery. The ocular pharmacokinetics of topical indomethacin suspension has been studied in rabbits and the peak concentrations in aqueous were achieved within 30 min (Bucolo et al., 2011). Further pranoprofen was administered three times, 1 h before surgery could help maintain the intraoperative pupil diameter obviously. Therefore, in our study, pranoprofen eye drops were administered 1 h before surgery, which was also convenient and feasible for cataract patients. While the exact concentration in aqueous humor and the ocular pharmacokinetics of pranoprofen need to be further studied. In addition, pranoprofen, as one of NSAIDs, is a kind of propanoic acid with both hydrophilic and hydrophobic properties, and prescribed as anti-inflammation and pain relief therapy in clinical practice. NSAIDs are a group of chemically heterogeneous drugs with similar analgesic, antipyretic, and anti-inflammatory effects. All NSAIDs suppress prostaglandin (PG) synthesis by inhibiting COX-1 and COX-2 (Semeraro et al., 2015). Pranoprofen is mainly used for its anti-inflammatory effects, to prevent macular edema after cataract surgery. It has been reported that the preoperative administration of topical NSAIDs improves the maintenance of the intraoperative pupil diameter and reduces the discomfort after cataract surgery (Razmju et al., 2012; Zanetti et al., 2012; Faktorovich and Melwani, 2014). It is also well established that the administration of topical pranoprofen postoperatively significantly reduces the ocular pain and discomfort after PRK compared with the pain relief afforded by a placebo (Mohammadpour et al., 2011). In our study, the matching demographic and clinical characteristics of the two groups maximized the comparability of their pain perception, and the greater pain during sequential second-eye surgery was significantly reduced by the preoperative administration of NSAID eye drops. The satisfaction and cooperation of the patients were also improved.

When the interval between the first-eye and second-eye cataract surgery was 1 week or 6 weeks, the pain scores during second-eye surgery were much greater than those when second-eye surgery was performed after a different interval. Therefore, the preoperative administration of pranoprofen is especially strongly recommended after these two intervals. This finding also suggests that second-eye cataract surgery should be scheduled to avoid these two intervals.

In the 1-week subgroup, the aqueous MCP-1 levels and VAS_pain_ scores during second-eye surgery were significantly reduced by the preoperative administration of pranoprofen eye drops. Similarly, in the ECLE rat model, the MCP-1 concentration in the contralateral eye was greater in the 1-day and 3-day interval groups than in the other subgroups, and was reduced by the administration of pranoprofen. MCP-1 and perceived pain were significantly increased during second-eye cataract surgery compared with first-eye surgery in the control group. A linear regression analysis also suggested a positive correlation between MCP-1 and the VAS_pain_ scores during second-eye surgery. MCP-1 reportedly correlates strongly with pain severity in patients with arthritis or fibromyalgia (Cuellar et al., 2009), and was shown to interact with nociceptive sensory neurons in a rat model (Sun et al., 2006). MCP-1 was confirmed to be a pain-associated factor in our study. It has been reported that when C57 mice were unilaterally exposed to UVR-B, inflammatory cell infiltration (including macrophages and polymorphonuclear cells) was detected in the contralateral eyes of the mice. Therefore, a systemic inflammatory response may be induced by UVR-B exposure (Meyer et al., 2013). The acute phase of inflammation is characterized by the rapid influx of granulocytes and neutrophils, followed swiftly by monocytes, which mature into macrophages and affect the function of the resident tissue macrophages (Poveschenko et al., 2015). MCP-1, a pain-related cytokine, is produced by many cell types, especially monocytes and macrophages. Because it is an inflammatory cytokine, increased levels of MCP-1 are observed in many inflammatory diseases of other organs, including atherosclerosis and cancer (Kok et al., 2009; de Waard et al., 2010; Li and Tai, 2013). Consequently, the increased MCP-1 levels in our study are thought to correlate with the inflammatory response in the second eye, which was induced by the first-eye surgical procedure. The specific mechanism of pranoprofen in relieving pain in this study is unclear, but we tentatively attribute it to a direct effect on the inflammatory response via its inhibition of PG and macrophages, which play central roles in the generation of the inflammatory response. PGE2, the most abundant PG, is known to modulate the inflammatory response (Kawahara et al., 2015). The prostanoid receptor appears to play a proinflammatory role by regulating the production of inflammatory cytokines, such as interleukin 1β (IL-1β), IL-6, and MCP-1, and its deficiency promotes macrophage apoptosis. Other findings have demonstrated that the inhibition of PGE2 markedly reduces the inflammatory response (Stinson et al., 2014; Zhu et al., 2014). However, the specific pathway and the infiltration of inflammatory cells in the contralateral eye require further confirmation.

When the second-eye surgery was performed after intervals of 2–8 weeks, the MCP-1 in the second eye decreased and remained at a stable level. This indicated that during the resolution of inflammation, the granulocytes were eliminated, the macrophages and lymphocytes returned to normal preinflammatory numbers and phenotypes, and the concentrations of inflammatory cytokines decreased (Buckley et al., 2014). However, in the 6-week subgroup, the VAS_pain_ scores were greater during second-eye surgery in the control group, although they were reduced by the preoperative administration of pranoprofen eye drops. The specific pain mechanism, which may not involve MCP-1, is still unclear, but we hypothesize a delayed inflammatory response.

## Conclusion

Our results indicate that the preoperative administration of pranoprofen significantly reduces the severity of pain during sequential second-eye cataract surgery, especially when this surgery is performed 1 or 6 weeks after the first-eye surgery. We suggest that an MCP-1-associated inflammatory response occurs in the second eye 1 week after first-eye surgery, which induces greater pain during second-eye surgery, and that this pain can be reduced by the administration of pranoprofen.

## Author Contributions

YL designed and conducted the study. YZ, YD, and YJ collected and managed the data. YZ analyzed and interpreted the data and prepared the manuscript. YJ, XZ, and YL reviewed the manuscript. YZ, XZ, and YL approved the final manuscript.

## Conflict of Interest Statement

The authors declare that the research was conducted in the absence of any commercial or financial relationships that could be construed as a potential conflict of interest.
